# Challenges of the epidemiological and economic burdens associated with hypertension in middle income countries: evidence from Mexico

**DOI:** 10.1186/s12889-015-2430-x

**Published:** 2015-11-06

**Authors:** Armando Arredondo, Silvia Magali Cuadra, Maria Beatriz Duarte

**Affiliations:** National Institute of Public Health-Mexico, School of Public Health, University of Montreal, Cuernavaca, QC H3T 1 J4 Mexico; National Institute of Public Health, Av Universidad 655, CP 62508 Cuernavaca, Mexico

**Keywords:** Epidemiological changes, Financial consequences, Hypertension, Health system, Users

## Abstract

**Background:**

In order to identify the challenges resulting from hypertension in a middle income country, this study has developed probabilistic models to determine the epidemiological and economic burden of hypertension in Mexico.

**Methods:**

Considering a population base of 654,701 reported cases of adults with hypertension, we conducted a longitudinal analyses in order to identify the challenges of epidemiological changes and health care costs for hypertension in the Mexican health system. The cost-evaluation method used was based on the instrumentation technique. To estimate the epidemiological changes for 2015–2017, probabilistic models were constructed according to the Box-Jenkins technique.

**Results:**

Regarding changes in expected cases for 2015 vs. 2017, an increase of 12 % is expected (*p* < 0.001). Comparing the economic impact in 2015 versus 2017 (*p* < 0.001), there is a 23 % increase in financial requirements. The total amount for hypertension in 2016 (US dollars) will be $6306,685,320 Of these, $ 2990,109,035 will be as direct costs and $ 3316,576,285 as indirect costs.

**Conclusions:**

If the risk factors and care models remain as they are currently in the health system, the financial consequences will have a major impact on the out-of-pocket users, following in order of importance, on social security providers and on public assistance providers.

## Background

In the middle-income countries (MICs), hypertension issues are leading public health challenges to the health system and to society. Hypertension is a health problem that requires an integrated approach. The increasing trend in the prevalence of hypertension in recent years has been constant. It seems that the increase in hypertension cases will continue [1].

In Mexico, the prevalence of hypertension increased from 26.6 % in 1993 to 31.8 % in 2012 [2]. The impact of this disease is evident not only on mortality, but also on morbidity and the quality of life. This morbidity, compared to other chronic diseases, represents an important burden for individuals and their families, as well as for the health system and society in general [3].

The observed and forecasted changes in the incidence of hypertension in adults will generate constant increases in the need for health services [4]. The observed changes represent a high economic burden for health systems, in the medium and long term [5].

To date, in middle-income countries, there are no detailed studies of the financial requirements for health services needed for hypertension in the next years [6]. Thus, having no knowledge of the costs, it is impossible to develop resource allocation patterns for a more efficient use of hypertension-related expenditures [7].

The constantly increasing costs of medical care and the unknown costs of ambulatory case management and hospital cases, justify the development and use of indicators for the increasing changes in the demand and costs of case management in the future [8].

The purpose of this study is to identify expected cases and financial requirements for hypertension during the 2015–2017 period. We highlight the implications of the economic impact of hypertension on the users’ pockets and on the providers of health services as one of the main challenges to be solved by health planners in resource allocation for public health interventions.

## Methods

An evaluative research was carried out, based on a longitudinal design to determine costs, epidemiological changes and financial requirements to deliver health care for hypertension during the 2015–2017 period. The three-year period was chosen for the study for several reasons: Projections with more than 3 years may generate uncertainty in the number of expected cases and hence are not recommended for strategic planning reasons; the health budget is revised, adjusted and approved for a three-year period by members of the Budget Commission of the legislative power; the inflationary consumer price index, applied to the direct health costs, is estimated by the Banco de Mexico, for consecutive periods every 3 years. Thus, to estimate indirect health costs under the human capital focus, it is preferable to limit the study to short term periods [9, 10].

Whereas analysis of economic impact relates to changes in the demand for services required for expected cases, case demand concerns the number of cases that request services and are under treatment and annual monitoring at each institution of the Mexican health system. The studied institutions belong to the public health sector of the Mexican health system: SSA (services for the uninsured population) and IMSS/ISSSTE (health care services for the insured population). (For more details on the health system in Mexico and the institutions included in the study, see Appendix 1). The basic protocol of this project was reviewed and ethically approved by the Committee on Health Research of the National Council of Science and Technology [11].

The demand for expected cases was estimated based on the following methodological steps [12]:

- Step 1. Modeling: a) identification of the tentative model for the time series, for use in the prediction, b) checking the quality of the information and number of observations, and c) analysis of the autocorrelation of the historical observations.
- Step 2. Estimation: a) determination of the estimates of the parameters, using the least squares criterion, b) application of an iterative procedure searching for a sum of squares function, previously specifying the preliminary estimates of the unknown parameters.
- Step 3. Diagnostic check: a) adequacy test, after the models were fitted to the data, b) analysis of the difference between the observed and expected results, c) application of the Box-Pierce chi squared.
- Step 4. Prediction: a) selection and design of the definitive model, b) data processing, c) prediction of the future values of the time series.

The time series analysis was based on the total annual cases observed in the last 18 years in each studied institution. In our study we delimit the record to the 1996–2013 period, because before 1996, quality standards in the records were not high. Before 1996, there were no well trained personnel for the differentiated recording of cardiovascular diseases according to the International Classification of Diseases. The population base of the study included 654 701 cases of hypertension, which were medically diagnosed, reported in the year prior to the study (2014). This information was obtained from the health impairment statistics bulletin of the National Health System [13]. This is anonymous information that is used for the purpose of analysis.

Direct costs refer to the average medical costs of annual case management per patient and for 3 main complications. Information on health care services was obtained from the management of standardized cases, adjusted by type of institution. The standardization and adjustment by type of institution, were performed with the application of a discount rate of 2 % annually, based on the cost of annual average case handling and cost of inputs (human resources, medicines, healing materials, utilities, furniture and medical equipment) by type of institution. In order to control the costs attributable to hypertension, the cost-evaluation method was designed according to an instrumentation technique that identified production and supply functions for each case management validated by consensus of experts from each institution. For each function production (medical visit, hospitalization, diagnostic studies, etc.) to be evaluated, management of the average case was defined, based on the disease’s natural history and the results from a shadow study of all stages of the process which patients go through when requesting health services for hypertension [14].

The indirect costs were determined using the human capital model developed for chronic diseases in Latin America [15]. This model was adjusted and designed to include three categories of costs attributable to hypertension: premature mortality, permanent and temporal disability.

To calculate the financial consequences of changes in demand by type of institution, in optimal scenarios, controlling the effects of inflation, an inflationary index projected to 2015–2017 was developed and applied, based on the last *Banco de Mexico* price index for consumers [16]. In order to have an overview of the annual direct and indirect costs of hypertension by item, the analysis was restricted to 2016, since it corresponds to one-half of the projected time period.

## Results

The resulting model for estimating the expected cases of hypertension was a model with average movement operator of order 1. The estimated model of prognosis of demand refers to the 2015–2017 period. Historical data were annual numbers of hypertension cases reported over the 1996–2013 period. These numbers of cases, denoted as Y_1_, Y_2_,…,Y_18,_ showed an increasing trend throughout the study period resulting in a slope coefficient of the trend analysis that was significantly different from zero (t = −.12). The data also showed a seasonal pattern with an increasingly higher peak each year, which coincides with the seasonal factor. Data were log-transformed to reduce the observed asymmetry. The natural logarithm of the number of cases is denoted by Y_1_,Y_2_,…,Y_18_.

One can see that the series is non-seasonal due to the slow decrease in the self-correlation function. After applying several seasonal and non-seasonal transformations, we inferred that the seasonal time series can be explained by the transformation: Z_t_ = Y_t_ - Y_t-1_ - Y_t-12_ + Y_t-13, having_ one seasonal and one non-seasonal effect. Upon analyzing the self-correlation function, it seems that it cuts the borderline of statistical significance of zero difference after lag 12. Some peaks are observed for smaller lags, indicating the need to include a seasonal average movement operator with some self-regressive operator and/or average movement operator of a smaller order. Based on the analysis of both functions, the following models are proposed:

MODEL 1. Seasonal average movement operator, order 1

$$ {\mathrm{Z}}_{\uptau}-\updelta +{\upvarepsilon}_{\uptau}-{\uptheta}_{1,13}\kern0.5em {\upvarepsilon_{\uptau}}_{-12} $$

MODEL 2. Seasonal average movement operator, order 1, and non-seasonal average movement operator, order 1

$$ {\mathrm{Z}}_{\uptau}-\updelta +{\upvarepsilon}_{\uptau}-{\upbeta}_{1,13}{\upvarepsilon_{\uptau}}_{-12}-{\uptheta}_1\kern0.5em {\upvarepsilon}_{\uptau}+{\upbeta}_1\kern0.5em {\upbeta}_{1,12}\kern0.5em {\upvarepsilon}_{\uptau -13} $$

MODEL 3. The same as model 2, but including the mean

MODEL 4. Seasonal self-regressive operator, order 1

$$ {\mathrm{Z}}_{\uptau}=\updelta +{\upphi}_{\kern0.5em 1,12}\kern0.5em {{\mathrm{Z}}_{\uptau}}_{-12} $$

MODEL 5. Non-seasonal self-regressive operator, order 1 and seasonal self-regressive operator, order 1

$$ {\mathrm{Z}}_{\uptau}=\updelta +{\upphi}_{1,12}\kern0.5em {\mathrm{Z}}_{\uptau}-12+{\upphi}_{\kern0.5em 1}\kern0.5em {\mathrm{Z}}_{\uptau \hbox{-} 1}-{\upphi}_{\kern0.5em 1}\kern0.5em {\upphi}_{\kern0.5em 1,13}\kern0.5em {\mathrm{Z}}_{\uptau \hbox{-} 13} $$

To select among several possible models, it was necessary to estimate each ones’ parameters and to examine its properties. Model 1, which included only the seasonal average movement operator, fits the data’s logarithms with reasonably good results and a self-correlation different from zero in lag 1, thus indicating that the model may be improved by adding a self-regressive and/or non-seasonal average movement operator.

Besides the seasonal average movement operator, model 2 included the non-seasonal average movement operator, which showed quite significant improvement in the standard deviation value that decreased from .4798 to .38007, and no nonzero self-correlation of residuals. The discussion on model 3 is carried out after discussing predictions. Models 4 and 5, which included seasonal and non-seasonal self-regressive operators, were also tested and were discarded because they did not meet adequacy conditions (substantially greater values of the Box-Pierce Chi-square and of standard deviation than in the other two models). The model that adequately depicted the series to estimate hypertension cases was the following:

$$ {\mathrm{Z}}_{\uptau}=\updelta +{\upvarepsilon}_{\uptau}-{\upbeta}_{1,13}\kern0.5em {\upvarepsilon}_{\uptau -13}-{\uptheta}_1\kern0.5em {\upvarepsilon}_{\uptau -1}+\kern0.5em {\uptheta}_1\kern0.5em {\uptheta}_{1,12}\kern0.5em {\upvarepsilon}_{\uptau -13} $$

with the prediction equation:

$$ {\upgamma^{+}}_{\uptau}=\updelta +{\upgamma}_{\uptau -13}+{\upbeta}_{1,12}\kern0.5em {\upvarepsilon}_{\uptau -13}-{\uptheta}_{1\kern0.5em }{\upvarepsilon}_{\uptau -1}+{\uptheta}_{1\kern0.5em }\kern0.5em {\uptheta}_{1\kern0.5em ,12}{\upvarepsilon}_{\uptau -13}+{\upvarepsilon}_{\uptau} $$

Therefore, the proposed model was model 3, which besides average movement and seasonal average movement operators, includes the mean estimator and variables mentioned in the methodology section. The outcomes of this model were better than those obtained using model 2 (lower standard error) whereas the t value for the mean estimator indicates that the trend is negative and significantly different from zero (see Table 1).

**Table 1:** Statistical results of models 2 and 3

Variables	Model 2	Model 3
Number of nonseasonal differences	1	1
Number of seasonal differences	0	0
Number of Parameters	2	3
Estimator A	.2579 (2.43)	.7689 (4.01)
Estimator B	-.2723 (−2.54)	.5536 (2.23)
Estimator C	.8599	.9123
Box-Pierce Chi-square	22.25	14.67
Standard deviation	24.22	26.9
Nonzero correlations	0	0

The findings of expected hypertension cases during the study period have constant incremental trends in the population served at the 3 major health institutions in Mexico (see Table 2). These trends are stronger in the case of insured population showing an increase for 2015–2017 (*p* < 0.001). Adding up the cases that increase annually by type of institution, the result is 72895 cases for the insured population (IMSS and ISSTE) vs 38109 cases for the uninsured population (SSA).

**Table 2:** Expected cases for hypertension in Mexico to the period 2015–2017 by type of institution

Type of institution	Number of cases per year			
	2015	2016	2017	*p* value
SSA	291904	306896	330013	<0.001
CI^a^	(284048–299760)	(298896–314896)	(321501–338525)	
IMSS	324447	348903	374603	<0.001
CI^a^	(315782–333112)	(339591–358215)	(366890–382316)	
ISSSTE	133350	144233	156089	<0.001
CI^a^	(127669–139061)	(137383–151083)	(149589–163589)	

Source: Arredondo et al. (2014) [17] Costos y consecuencias financieras del cambio en el perfil epidemiológico en México. INSP–University of Montreal-International Development Research Centre, 1999–2013. Update of probabilistic models, January 2015

Box–Pierce statistical test (*P* < 0.001)

*SSA* Ministry of Health, *IMSS* Mexican Institute for Social Security, *ISSSTE* Institute for Social Security and Services for State Workers

^a^95 % CIs

The results on case management costs and on financial requirements to satisfy the demand for hypertension services in the coming years (see Tables 3 and 4) are new, pertinent and relevant evidence for an efficient management of hypertension in the near future in Mexico. Table 3 describes the main results of the expected costs and financial requirements for each year of the study (2015–2017) for the entire health system. Table 4 describes the expected economic burden only for 2016 as a cut-off point, differentiating costs by type of cost, type of institution and type of complication.

**Table 3:** Cost from epidemiological changes of hypertension expected for the years 2015, 2016, 2017 in Mexico (in US$)

Item cost		Year		
	2015	2016	2017	*p* value
Direct costs				
Consultations/diagnosis	342,503,700	358,813,400	391,432,800	<0.001
Drugs	399,587,467	418,615,441	456,671,390	<0.001
Hospitalization	313,961,277	328,911,814	358,812,888	<0.001
Nephropathy	988,978,836	1,036,073,067	1,130,261,527	<0.001
Nonfatal myocardial infarction	449,535,634	470,942,093	513,755,010	<0.001
Nonfatal stroke	359,628,074	376,753,221	411,003,513	<0.001
Total direct costs	2854,194,988	2,990,109,035	3,261,937,129	<0.001
Indirect costs				
Mortality	474,872,870	497,485,864	542,711,851	<0.001
Permanent disability	2342,709,670	2,454,267,274	2,677,382,480	<0.001
Temporary disability	348,240,277	364,823,148	397,988,888	<0.001
Total indirect costs	3,165,822,818	3,316,576,285	3,618,083,220	<0.001
TOTAL COSTS	6,020,017,806	6,306,685,320	6,880,020,349	<0.001

Source: Arredondo et al. (2014) [17] Costos y consecuencias financieras del cambio en el perfil epidemiológico en México. INSP–University of Montreal-International Development Research Centre, 1999–2009. Update of probabilistic models, January 2015

Box–Pierce statistical test (*P* < 0.001)

Exchange rate: January 2015, 1 US$ = 14.85 Mexican $

**Table 4:** Direct, indirect and total costs (in US$) for health care service providers and users attributable to hypertension expected for the year 2016 in Mexico

Item of cost					
	SSA^a^	IMSS	ISSSTE	Users	TOTAL
Direct costs					
Consultations/diagnosis	45,733,525	87,930,959	35,154,160	182,994,757	358,813,400
Drugs	61,522,443	102,586,116	41,013,184	213,493,698	418,615,441
Hospitalisation	48,339,063	80,603,376	32,224,645	167,744,730	328,911,814
Nephropathy	152,268,051	253,900,637	101,507,633	528,396,746	1,036,073,067
Nonfatal myocardial infarction	69,212,750	115,409,380	46,139,832	240,180,130	470,942,093
Nonfatal stroke	55,370,201	92,327,505	36,911,866	192,143,650	376,753,221
Total direct	439,446,033	732,757,971	292,951,320	1,524,953,711	2,990,109,035
Indirect costs					
Mortality	73,130,538	121,884,230	35,752,658	253,717,405	497,485,864
Permanent disability	360,777,319	601,295,532	240,518,212	1,251,676,210	2,454,267,274
Temporary disability	53,629,060	89,381,768	48,753,692	186,059,662	364,823,148
Total indirect	487,536,917	812,561,530	325,024,561	1691,453,277	3316,576,285
Total costs	926,982,959	1545,319,501	617,975,882	3216,406,987	6,306,685,320

Source: Arredondo et al. (2014) [17] Costos y consecuencias financieras del cambio en el perfil epidemiológico en México. INSP–University of Montreal-International Development Research Centre, 1999–2009. Update of probabilistic models, January 2015

Exchange rate: January 2015, 1 US$ = 14.85 Mexican $

^a^ *SSA* Ministry of Health, *IMSS* Mexican Institute for Social Security, *ISSSTE* Institute for Social Security and Services for State Workers, *Users* pocket payment

Comparing the economic impact in 2015 with the forecast for 2017 (*p* < 0.001), a 21 % increase in financial requirements has been forecasted (see Table 2). The total cost for hypertension in 2016 is forecasted to be US $6306,685,320 (see Table 3). This includes $2990,109,035 in direct costs and $3316,576,285 in indirect costs. The total costs for each institution are: $926,982,959 for the Ministry of Health, serving the uninsured population; $2163,295,383 for the insured population (the Mexican Institute for Social Security, and the Institute for Social Security and Services for State Workers); and $3216,406,987 for the out-of-pocket users. Nephropathy is the largest single contributor to the total cost of overall management ($1036,073,067). This is followed by the costs of treating nonfatal myocardial infarction ($470,942,093) and nonfatal stroke ($376,753,221).

The overall contribution of direct costs was 47 % of total costs and indirect costs were 53 %. With regard to the four categories of estimated direct costs, results were: consultations and diagnoses, 12 %; drugs, 14 %; hospitalizations, 11 %; and complications, 63 %. Distribution of total costs of complications was as follows: nephropathy, 55 %; nonfatal myocardial infarction, 25 %; and nonfatal stroke, 20 %. Results for the three estimated categories of indirect costs were: mortality costs, 15 %; costs of permanently disabled patients, 74 %; and costs of temporarily disabled patients, 11 % (see Table 3).

The institution for the insured population was found to have the highest average direct costs per managed case, as well as the highest economic impact of global management of hypertension for 2015–2017. Financial requirements for health care services for hypertension represent 19.5 % of the total budget assigned to the uninsured population, and 12.5 % of that allocated to the insured population.

## Discussion

This study addressed the need to generate information that is necessary, relevant and pertinent, for a more strategic planning of public health actions that are oriented towards a better management of chronic diseases such as hypertension. The estimation of expected cases and financial requirements for each year of the studied period and for each of the main institutions of the health system in Mexico represents one of its main contributions.

The finding related to the high contribution of patients and families to the total expenditure for hypertension in Mexico, is a fact that was unknown for this indicator. The constant increase in both epidemiological changes and the economic burden of hypertension, represents a very important finding that can be applied to the analysis of this problem in middle-income countries like Mexico, as well as in other Latin American countries, because of the great similarity of the health systems and epidemiological changes in chronic diseases in all these countries [17].

With respect to the differences in expected costs and financial requirements for the insured health subsystem vs. the uninsured subsystem, the differences in these results are mainly due to 3 reasons: The costs of inputs for social security institutions are higher, the population seeking care for hypertension is greater in uninsured care centers and because of differences in standards of quality of care and input mix in each institution.

There are also several studies of epidemiological trends in recent years but in very general terms, they do not specify the disease or the expected epidemiological trends for the expected cases in the future. Our results have similarities, in terms of constant incremental pressure trends, with other case studies published by different authors [18, 19], particularly with hypertension results of the last national health survey conducted in Mexico in 2013 [20]. Indeed, a tendency is expected for a constant increase in the required health care services and in costs, although the increase is greater for the insured population than for the uninsured population.

On the other hand, there is much talk about the high health expenditures of patients in MICs, but the exact measurement of expenditures by families and patients with chronic health conditions like hypertension, is not being considered. Our results certainly provide information about the high health expenditure by patients, attributable to hypertension.

In addition to some problems listed in the methods section, this study also suffers several more external limitations. First, on the epidemiological analysis of the frequency of cases, we only included cases of hypertension and co-morbidity of 3 main complications that required health services for diagnosis, treatment and control, in major health institutions in Mexico. In this sense, the analysis does not include cases of patients ignoring hypertension symptoms, or even knowing they are ill but cannot have access to health services for different reasons. Second, the main limitation of the time series analysis, using the Box-Jenkins method, is the great number of reports that are required, having high quality information on observed cases (15 years minimum), for a rigorous estimate of expected cases in future years. Our study covers an 18 year period (1996–2013), since before 1996, quality standards in the records were not high enough; However, there may be important limitations when trying to replicate our forecasting model in other middle income countries where the quality of the information may not be good enough in terms of the minimum number of annual observations that is required.

With respect to indirect costs, another limitation of this study, based on the human capital focus, is the possible overestimation of indirect costs, attributable to temporary disability, when friction costs are not determined. In the short-medium term, the indirect costs could be overestimated due to the fact that the patients may compensate for the loss of production in the long term, if they return to their job, or their work may be carried out by their co-workers. In the long term, the indirect costs could be zero if the patient improves and returns to his/her job, or else if an unemployed worker took his/her place at work after a “friction period,” in which case, costs must be included that relate to searching for and training the unemployed worker in his/her new position; this depends on the unemployment rate and disability work policies, such as those proposed by some authors [21]. However, in our study, indirect costs were limited to a 3-year period and it is not the objective of our study to disaggregate the effect of other variables.

## Conclusions

This study estimated the changes in the number of expected hypertension cases during the 2015–2017 period, in a middle income country. Direct and indirect costs were also identified for the management of an average hypertension case and its 3 main complications. The results are proposed as relevant information for decision-making in the planning and allocation of resources for health services for chronic diseases in Mexico. This is an example of what is happening in the management of hypertension in middle income countries.

The study’s results also reveal important evidence of the economic burden for the users’ pockets coming from hypertension management. In this sense, and in the context of the health reforms in MICs such as Mexico, aspects such as equity and access to health services are still an unresolved challenge.

## Appendix 1: Some considerations on Mexico’s health system

At their inception, in 1942, health policies in Mexico were put into effect by municipal actions oriented by the Consejo Superior de Salubridad (Higher Council for Health). These actions proved to be clearly deficient. An office of the Federal Executive had to be created, with sufficient power to allocate resources and regulate activities to control epidemics and improve urban sanitation. The Departamento de Salubridad (Department of Health) was created, supported by the Consejo de Salubridad General (Council for General Health) which served, along with legislative agencies, as the advisory and managing bodies to issue specific health interventions. Until 1929, this policy allowed for the implementation of Health Cooperative Units (primary health centers) working with states and municipalities.

During the 30’s, health policies that had been set during the revolutionary period (1910–1920) were still followed. In addition, a new health care model was created: joint health services offered by the government, agricultural development banks and peasants endowed with vast land extensions. Under this new policy, the Department of Health worked to start and maintain intensive care and curative care services. The new health policy became quite dynamic on account of government support for health services for workers.

The foundation of the present health system can be traced to 1943, the year in which IMSS and the SSA were established to convey tripartite contributions (state, enterprises and workers’ funds) to support the industrial development of the main cities and the public supply of comprehensive services. This model prevailed almost worldwide; its technical core was drawn from the International Labor Organization. The SSA arose from the fusion of the Secretaría de Asistencia (Welfare Secretariat) and the Departamento de Salubridad (Department of Health). Its healthcare mission was extended to provide more comprehensive care for the population left unprotected by social security, which included the majority of peasants, the unemployed, and informal economy workers. SSA was also in charge of launching massive campaigns to control epidemics and specific health problems. An additional advancement of this period was the creation of ISSSTE in 1959, which brought together the diversity of pension and fringe benefit systems for state workers.

Presently, according to data from the 2003–2018 National Health Program, the Mexican public health system provides care for 90 % of the population and is mainly formed by the three health institutions which were included in this study. It is important to highlight the fact that IMSS provides services for the insured population and covers 45 % of the population, ISSSTE serves the insured population of state workers and covers approximately 5 % of the population. Finally, since last years, the SSA in the context of universal coverage reforms, implemented as the main strategy program ‘Seguro Popular’, a program to cover the uninsured population, which is 40 % of the population. The remaining 10 % are served by the private health provider.

## Abbreviations

MICs: Middle income countries
SSA: Ministry of Health
IMSS: Mexican Institute for Social Security
ISSSTE: Institute for Social Security and Services for State Workers
